# Development of the Pre-gnathal Segments in the Milkweed Bug *Oncopeltus fasciatus* Suggests They Are Not Serial Homologs of Trunk Segments

**DOI:** 10.3389/fcell.2021.695135

**Published:** 2021-08-06

**Authors:** Oren Lev, Ariel D. Chipman

**Affiliations:** Department of Ecology, Evolution and Behavior, Alexander Silberman Institute of Life Sciences, The Hebrew University of Jerusalem, Jerusalem, Israel

**Keywords:** insect, arthropod, evolution, segmentation, evo-devo, head, GRN evolution, gene network analysis

## Abstract

The three anterior-most segments in arthropods contain the ganglia that make up the arthropod brain. These segments, the pre-gnathal segments (PGS), are known to exhibit many developmental differences to other segments, believed to reflect their divergent morphology. We have analyzed the expression and function of the genes involved in the conserved segment-polarity network, including genes from the Wnt and Hedgehog pathways, in the PGS, compared with the trunk segments, in the hemimetabolous insect *Oncopeltus fasciatus*. Gene function was tested by manipulating expression through RNA interference against components of the two pathways. We show that there are fundamental differences in the expression patterns of the segment polarity genes, in the timing of their expression and in the interactions among them in the process of pre-gnathal segment generation, relative to all other segments. We argue that given these differences, the PGS should not be considered serially homologous to trunk segments. This realization raises important questions about the differing evolutionary ancestry of different regions of the arthropod head.

## Introduction

Arthropods are a hyper-diverse animal phylum characterized by both high species numbers and exceptional biomass. One of the reasons for their immense success is believed to be their segmented body plan, which provides high evolvability *via* a modular organization (Chipman and Edgecombe, 2019).

Segments are repeating body units along the anterior to posterior body axis, which form in a developmental process known as segmentation. Although the specifics of the segmentation process vary among species (Peel et al., 2005; Nagy and Williams, 2020) and can even vary within an individual embryo (Stahi and Chipman, 2016; Auman et al., 2017), core aspects of the process are highly conserved, and the segments are generally accepted to be homologous between all arthropods and serially homologous within an individual (Minelli and Fusco, 2013; Scholtz, 2020). This serial homology is usually taken to mean that they are patterned using a shared and conserved developmental process, namely, the aforementioned segmentation.

Segmentation is often described as a hierarchical process, based on what is known from the *Drosophila* segmentation cascade, and the different stages of the process are named after the stages in the *Drosophila* mode. Most relevant to comparative analyses of segmentation are the genes of the two last stages: pair-rule genes and segment-polarity genes (Peel et al., 2005; Clark et al., 2019; Chipman, 2020). The pair-rule genes are expressed in a two-segment periodicity in *Drosophila* and in many other holometabolous insects. Their ancestral role is probably to generate the preliminary repeated pattern that is at the base of segmentation (Chipman, 2020). They often interact in a complex gene regulatory network (GRN), the details of which vary among species (Damen et al., 2005; Choe et al., 2006; Green and Akam, 2013; Clark, 2017; Reding et al., 2019). Segment-polarity genes are expressed in segmental stripes in *Drosophila* and in all other arthropods studied to date. Their role seems to be defining segmental (or parasegmental) boundaries. In contrast with the variable pair-rule GRNs, the segment-polarity genes function as a highly conserved GRN with similar interactions among and within all studied arthropods (Nüsslein-Volhard and Wieschaus, 1980; Von Dassow et al., 2000; Peel et al., 2005). The segment-polarity GRN is based upon the interactions of two signaling pathways, Hedgehog and Wnt, and the paralogous transcription factors Engrailed and Invected. It is unclear whether Engrailed and Invected have redundant functions to each other (Peel et al., 2006), but they have similar expression patterns in all known cases. Later identity of the segments is conferred by Hox genes, probably together with other genes (Auman and Chipman, 2017).

During development, segments are grouped into functional body units called tagmata. The organization of segments into tagmata varies among different arthropod clades, e.g., insects have three tagmata: head, thorax, and abdomen, while chelicerates have two: prosoma and opisthosoma. There is evidence to suggest that the differences between tagmata may have an early developmental basis (Chipman and Edgecombe, 2019).

The embryonic insect head can be divided into two regions: the anterior procephalon and the posterior gnathocephalon, which can be clearly distinguished based on their shape during the germband stage. Indeed, these regions have been referred to as separate tagmata (Akam et al., 1988; Minelli, 2001). The developing segments of the gnathocephalon are similar in size and shape to the thoracic segments that lie posterior to them. They develop to give rise to the three segments that carry the adult mouthparts: the mandibles, maxillae, and labium (Posnien et al., 2010). The embryonic procephalon makes up the head-lobes, an enlarged region without clearly distinguishable segments, which develops to give rise to three adult segments: the ocular, antennal, and intercalary segments, each of which has a dorsal ganglion. The three ganglia together make up the tri-partite brain. Because these three segments lie anterior to the limb-bearing gnathal segments in insects, we refer to them from here on collectively as the pre-gnathal segments (PGS). For simplicity, we will refer to all segments posterior to the PGS as trunk segments. There is mounting evidence that the PGS are homologous among all arthropods and share a deep evolutionary history (Ortega-Hernández et al., 2017). When discussing them in a general arthropod comparative framework, and not only in insects, they are usually referred to by the names of the ganglia they carry, namely (from anterior to posterior) the protocerebral, deutocerebral, and tritocerebral segments.

It has long been known that the PGS are developmentally distinct from other segments (Rogers and Kaufman, 1997). One significant difference between the PGS and the rest of the body segments is that pair-rule genes are not expressed in them during early development (Rogers and Kaufman, 1997; Posnien et al., 2010). In addition, in *Drosophila*, there is a group of interacting “head gap genes” that pattern the PGS and the mandibular segment (Crozatier et al., 1996, 1999; Wimmer et al., 1997). Finally, the later acting Hox genes, which are involved in conferring morphological identity to specific segments, are only expressed in the tritocerebral segment and not in the anterior two PGS.

The segment polarity genes are expressed in the PGS, but with some variations relative to what is found in other segments. Most notably, the timing of segment polarity gene expression is different: e.g., the segment polarity gene *engrailed* (*en*) is first expressed in the mandibular segment during the germband stage, even though the PGS are already patterned at that stage (Patel et al., 1989; Brown et al., 1994; Stahi and Chipman, 2016).

Furthermore, in several cases, *hedgehog* (*hh*) is expressed as a single domain, lying anterior to future segmental expression, that splits once or twice to give rise to three stripes, each marking a different one of the anterior segments. In the spider *Parasteatoda tepidariorum* and the centipede *Strigamia maritima*, *hh* expression splits twice, from the anterior stripe or the posterior stripe, respectively (Pechmann et al., 2009; Kanayama et al., 2011; Hunnekuhl and Akam, 2017). In the millipede *Glomeris marginata* (Janssen, 2012), the beetle *Tribolium castaneum* (Farzana and Brown, 2008) and the cricket *Gryllus bimaculatus* (Miyawaki et al., 2004), *hh* expression splits only once to give the protocerebral and deutocerebral expression stripes, with a third expression domain appearing later, marking the tritocerebral segment. Work in the model species *Drosophila melanogaster* showed unexpected interactions and expression domains of the different segment polarity genes in the anterior head segments compared to the body segments. For example, *hh* and *wingless/wnt1* have independent expression not involving *engrailed* expression (Gallitano-Mendel and Finkelstein, 1997). The authors of this work focused on relatively late expression, at a stage when the PGS had already undergone preliminary determination. The authors interpreted the differences in the PGS relative to other segments as being related to the different morphology of the structure formed from these segments, but did not consider differences in their earlier development.

While these previous studies have pointed out several unusual characteristics of the patterning of the PGS, there is no single species for which there exist both descriptive and functional studies in both early and late stages of the formation of the PGS. Furthermore, no previous study has attempted to reconstruct the sequence of gene interactions involved in generating these segments and to compare this sequence to that found in other segments. It is therefore difficult to identify when and where in development the significant differences between the PGS and the trunk segments first arise, or to draw evolutionary conclusions from these differences. We aim to fill this gap, using the milkweed bug *Oncopeltus fasciatus* as our chosen study organism for this analysis. *Oncopeltus* has been shown to be relatively conservative in many developmental processes (Chipman, 2017). Unlike *Drosophila*, components of the larval head are generated in full during early development of *Oncopeltus*, making it possible to study the development of all head structures in the embryo (Birkan et al., 2011). The earliest stages of PGS patterning occur during blastoderm segmentation, which has been studied extensively in *Oncopeltus*, is easy to access experimentally and is fairly well-understood (Stahi and Chipman, 2016). Furthermore, *Oncopeltus* is one of the few hemipteran species that have established molecular methods, making it an important model for studying development outside of the widely studied holometabolous radiation.

In this article, we provide a detailed descriptive and functional analysis of the early stages of the formation of the PGS in *Oncopeltus*. We show that at the level of the segment polarity genes, there are differences in the function and interactions of key genes, to an extent that the GRN patterning them cannot be considered to be the same network. Comparing our findings to what is known from the literature, we argue that the PGS in arthropods in general are patterned *via* a network that is different from that patterning the trunk segments.

## Materials and Methods

### Animal Culture and Egg Collection

*Oncopeltus fasciatus* cultures are kept in a temperature-controlled room at 25°C in plexiglass boxes. Each box contains several dozens of insects. To collect eggs, which are available year-round, cotton balls are placed in a box with sexually mature bugs, until a female lays a clutch on them or placed there for a pre-determined window of time. Eggs are kept in a 25°C incubator until they reach the desired stage of development.

### Antisense Digoxigenin-Labeled Probes and dsRNA Preparations

The primers for the antisense Digoxigenin (DIG) labeled RNA probes and for the dsRNA for parental RNA interference were designed from the published *O. fasciatus* genome (NCBI accession number: PRJNA229125) for all relevant genes using the bioinformatics software Geneious (Kearse et al., 2012).

Both probes and dsRNA were made using Sigma-Aldrich T7 RNA polymerase and buffer. Probes were made with Sigma-Aldrich Digoxigenin-labeled ribonucleotides (RNA). dsRNA was made using ribonucleotides from Lucigen.

### RNA Interference Using dsRNA Parental Injections

Virgin females are sedated with CO_2_ gas and injected with about 3–5 μL of dsRNA at an average concentration of 2 (±0.5) mg/mL of respective mRNA in the ventral abdomen. The injected females recover overnight. The following day, each female is introduced to 1–2 adult males and placed in a box with cotton balls. Egg collecting starts from the third day of egg-laying.

### Egg Fixation and Peeling

*Oncopeltus fasciatus* eggs are submerged in tap water and boiled for three minutes and then immediately placed on ice for at least 5 min. The water is then replaced with a 50/50% mixture of 12% paraformaldehyde in PBS-T (phosphate buffered saline + tween) / heptane and put in a shaking wheel for primary fixation. All liquids are then removed, followed by several washes with 100% methanol. The embryos can be kept at −20°C. To prepare for *in situ* hybridizations, *O. fasciatus* eggs are peeled and post-fixed in 4% paraformaldehyde in PBS-T for 90–120 min in a rotating plate.

### *In situ* Hybridization Staining

Fixed embryos are stepwise transferred to PBS-T solution and placed in a hybridization buffer for at least 1 h in 60°C. Antisense probes (Digoxigenin labeled RNA) are added to the buffer for a final concentration of 1 mg/mL probe/hybridization solution, and incubated at 60°C overnight. The embryos are washed several times with hybridization buffer to remove excess probes, and then PBS-T before being transferred to 10% normal goat serum/PBS-T (blocking) solution for epitope blocking. AP-conjugated antibodies against Digoxigenin are added to a final concentration of 1:2500 antibodies/blocking solution, and the embryos are placed at 4°C overnight.

The embryos are then washed with PBS-T and transferred to a staining solution. We stain the embryos until we observe noticeable staining or until the embryo gains a purple tint, suggesting non-specific staining. We transfer the embryos stepwise to 70% glycerol/PBS-T and keep them at 4°C. Stained embryos are then counterstained with DAPI or with Sytox-green.

### Microscopy

*Oncopeltus fasciatus* pictures were taken using an AZ100 stereoscope (Nikon) with Nikon Digital Sight DS-Fi1 camera.

### Primers

Sequences for all primers used to prepare probes and dsRNA can be found in Supplementary File 1.

## Results

### Early Wild-Type Expression of Components of the Segment-Polarity Gene Network

**FIGURE 1:**
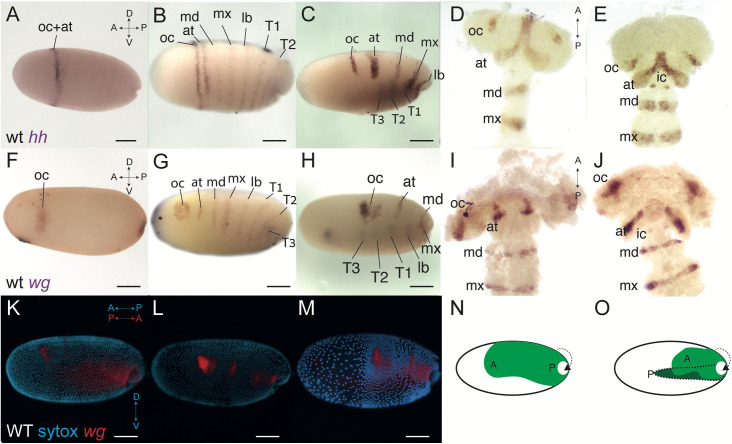
Wild-type expression of *hedgehog* (*hh*) and *wingless* (*wg*) in *Oncopeltus*, before and after blastoderm invagination. **(A)** Expression of *hh* at 32 h after egg laying (hAEL), late blastoderm stage, is as a ring in the anterior third of the embryo. **(B)** At around 36 hAEL, the *hh* anterior expression ring splits into two stripes corresponding to the presumed ocular and antennal segments. Six additional stripes of expression appear, corresponding to the presumed gnathal and thoracic segments. **(C)** Embryo at mid-invagination, ∼42 hAEL. The two anterior stripes are fully separated. Thoracic segments have already invaginated and expression in them can be seen through the yolk. **(D,E)** Germband embryos, ∼48–52 hAEL. In the later germband embryo, ∼52 hAEL **(E)**, two dots of *hh* expression appear in the presumed intercalary segment, which are not seen in the earlier embryo **(D)**. **(F)** Expression of *wg* at ∼32 hAEL is located to an anterior stain and a posterior stain marking the future invagination site. **(G)** At ∼36 hAEL seven stripes of expression appear simultaneously, corresponding to the presumed antennal, gnathal, and thoracic segments. **(H)** Embryo at mid-invagination, ∼42 hAEL. This embryo is slightly older than the one in **C** as evidenced by the extent of invagination. **(I,J)** Germband embryos, ∼48–52 hAEL. In the later germband embryo, ∼52 hAEL **(J)**, two dots of *wg* expression appear in the presumed intercalary segment, which cannot be seen in the earlier embryo **(I)**. **(K,M)** The process of invagination from ∼36–42 hAEL. Nuclei stained by SYTOX in cyan and wild-type *wg* expression in red. When invagination begins **(K)**, the blastoderm is still mostly uniform. At mid-invagination **(L)** most blastoderm cells migrate dorsally, with the anterior cells, corresponding to the future head, condensing to give a head lobe. At late invagination **(M)** the head lobe is distinct. At this stage, the anterior-posterior axis of the egg is opposite to that of the invaginating embryo. **(N,O)** Illustration of the invagination process. “A” marks the embryonic anterior and “P” the posterior. The portions of the embryo that have invaginated to give the germband are shown in darker green. Panel **(N)** corresponds to the embryo in **(L)** and panel **(O)** corresponds to the embryo in **(M)**. at, antennal segment; ic, intercalary segment; lb, labial segment; oc, ocular segment; md, mandibular segment; mx, maxillary segment; T1–T3, first to third thoracic segments. Axis labels: A, anterior; P, posterior; D, dorsal; V, ventral. All scale bars are 200 μm.

We started by looking at the expression of two of the segment polarity genes, *hedgehog* and *wingless*, from the earliest stages of formation of the PGS and up to the early segmented germband stage (Figure 1). Expression of *hh* begins in the mid blastoderm, approximately 30–32 h after egg laying (hAEL at 25°C). It is first expressed as a ring at the anterior third of the embryo (Figure 1A). Just before blastoderm invagination begins (∼36 hAEL) there are two distinct dynamics: a stripe appears abutting the anterior ring and gradually splits away from it, while six more stripes appear more or less simultaneously between the first ring and the invagination site (Figure 1B and Supplementary Figure 1).

Throughout invagination (∼36–48 hAEL) the expressions stripes of *hh* become narrower and dorsally restricted as the two anterior-most stripes fully separate (Figures 1C, D). Around 50 hAEL there is a new expression of *hh* in two dots (Figure 1E). Based on their relative position and shape, and based on previous work on blastoderm segmentation (Stahi and Chipman, 2016) we interpret the two stripes that emerge from the gradual splitting of the initial ring as representing the ocular and antennal segments, and the six simultaneously appearing stripes as representing the three gnathal and three thoracic segments. Based on their location in the germband, the two late dots represent expression in the developing intercalary segment.

Blastodermal expression of *wg* has been previously described in *Oncopeltus* (Stahi and Chipman, 2016). However in previous work we did not follow expression into the germband stage, and we believe we therefore did not identify all of the expression domains correctly. We thus describe it again here. The earliest expression of *wg* appears in two domains: a posterior spot at the future invagination site, and an anterior irregular domain (Figure 1F). The anterior domain is slightly anterior and ventral to the *hh* ring that appears at the same time (Figure 1A). This domain [referred to by some authors in other species as the “head blob” (Liu et al., 2006)] expands and develops two lobes to give a heart shape (Figures 1G,H). At the same time, seven blastodermal segmental stripes appear simultaneously (Figure 1G). The six more posterior stripes extend from the dorsal side of the embryo to almost to the ventral side, and correspond to the gnathal and thoracic segments. The anteriormost stripe is shorter and corresponds to the antennal segment. This stripe was previously incorrectly identified as the intercalary stripe. Contrary to what we claimed in Stahi and Chipman (2016), the two anterior lobes of the “blob” never separate completely, and the heart shape continues until the germband stage, where it is found in the area of the head lobes corresponding to the ocular segment (Figures 1I, J). The actual expression of *wg* in the intercalary segment appears only in the germband, as two dots (Figure 1J), similar to the intercalary expression of *hh* (Figure 1E).

The shortening and thickening of the segmental stripes of *hh* and *wg* are due to the condensation of the germband and the head lobes that occurs during blastoderm invagination, as evidenced by staining for nuclei together with gene expression (Figures 1K–M). This condensation is also presented schematically in Figures 1N, O. During condensation, the cells that will form the head lobe move dorsally, to give two lobe-like structures, connected medially.

### Functions of the Segment Polarity Components in the Pre-gnathal Segments

To test the function of the Hedgehog and Wnt pathways in the anterior of the embryos, we used parental RNA interference (pRNAi) against positive and negative regulators of each pathway. A positive regulator will decrease the pathway’s activity when knocked-down, while a negative regulator will increase it. For the Hedgehog pathway, we chose *hedgehog* (*hh*) – encoding the single arthropod ligand that activates the pathway – and *patched* (*ptc*) – encoding the receptor, which downregulates Hedgehog-signaling when active. For the Wnt pathway, we chose *disheveled* (*dsh*) –which encodes an activating intracellular component of the pathway – and *shaggy* (*sgg*) – which encodes an enzyme that phosphorylates the second messenger β-catenin, thus leading to its degradation. We chose to not directly target either the ligand or the receptor in Wnt-signaling, since both have several paralogs that are at least partially redundant (Janssen et al., 2010; Murat et al., 2010).

**FIGURE 2:**
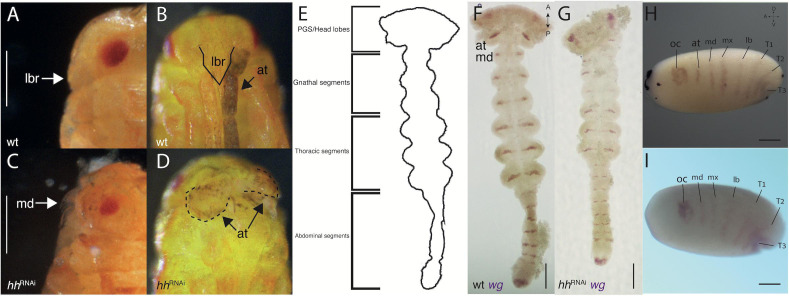
*hh*-RNAi phenotypes. **(A,B)** side and front view of a wild-type hatchling, the labrum (outlined with a solid line) covers the mandibles and the antennae lie to its sides. **(C)** A side view of a hatchling with a reduced labrum, exposing the mandibles following a *hh*-RNAi. **(D)** Front view of the same hatchling as **(C)** showing malformed stubby antennae (outlined with dashed lines). **(E)** Outline sketch of a germband stage embryo, showing the different regions (based on panel **F**). **(F)** Wild-type germband expression of *wg* in the mid germband stage. **(G)** *hh-*RNAi germband expression of *wg*, the antennal expression missing. **(H)** Wild-type expression of *wg* in the blastoderm (the same embryo from Figure 1G). **(I)** *hh-*RNAi expression of *wg* in the blastoderm, the antennal segmental stripe is missing. Abbreviations as in Figure 1. Axis labels: A, anterior; P, posterior; D, dorsal; V, ventral. All scale bars are 200 μm.

Reducing Hedgehog activity *via hh*^RNAi^ results in a consistent phenotype (Figure 2) – the reduction of PGS-related G structures: The eyes are smaller, and the labrum and antennae are reduced or not developed at all (Figures 2C, D compare with Figures 2A, B). Without the labrum, the hatchling mandibles and maxillae are exposed. The reduction is also visible when comparing WT *wg* expression in the head lobes (Figure 2F) to the *wg* expression of *hh*^RNAi^ germbands (Figure 2G). The segmental *wg* stripe representing the antennal segment is weak or non-existent in the head lobes during the germband stage, and the head lobes are misshapen and missing the most anterior region. The absence of the antennal *wg* stripe is clear from the first appearance of segmental stripes in the mid-blastoderm (Figure 2I compare with Figure 2H). There is no obvious effect in other regions of the body.

**FIGURE 3:**
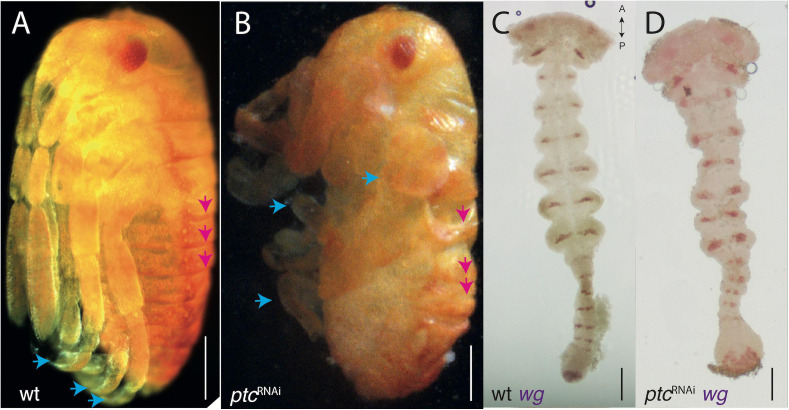
*ptc*-RNAi phenotypes. **(A)** Wild-type hatchling **(B)** *ptc*-RNAi hatchling of the same age with disrupted segmental boundaries (pink arrows mark the first three abdominal segments) and limb deformities (blue arrows). Compare with **(A)**. **(C)** Wild-type expression of *wg* in the germband (same embryo from Figure 2E). **(D)** *ptc-*RNAi expression of *wg* in the germband showing a normal head and mis-formed trunk segments. Axis labels: A, anterior, P, posterior. All scale bars are 200 μm.

Over-activating Hedgehog signaling through *ptc* knock-down has a different effect to *hh*^RNAi^, causing deformation of the trunk segments, legs becoming club-like and misshapen, but without any clear effect on the head (Figure 3). This is evident both in knock-down hatchlings (Figure 3B compare with Figure 3A) and in mounted germbands stained for *wg*, where the segment are somewhat laterally compressed and *wg* expression stripes are thicker (Figure 3D compare with Figure 3C). Prior to mounting and flattening the germband to a slide, the embryos are twisted relative to the straight WT germbands (not imaged). The early blastodermal expression of *wg* is not affected by over- or under-activating Hedgehog signaling (Supplementary Figures 2B, C). Late blastodermal expression is not affected by over-activation (not shown).

**FIGURE 4:**
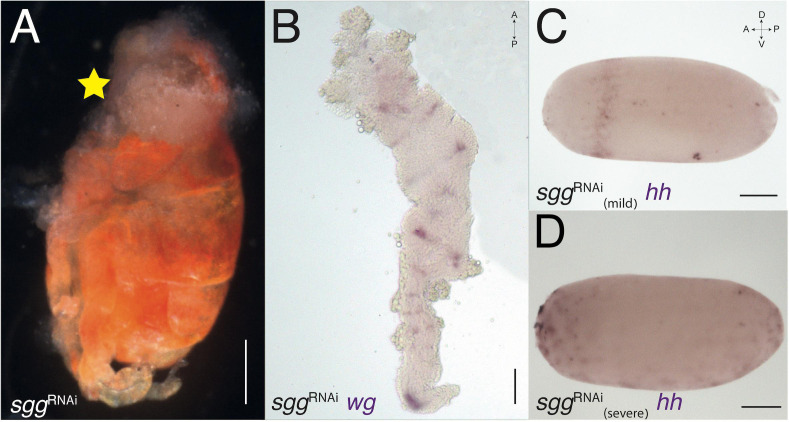
*sgg-*RNAi phenotypes. **(A)***sgg-*RNAi hatchling with no head (marked with yellow star) and a reduced abdomen (compare with Figure 3B). **(B)** Expression of *wg* in *sgg-*RNAi germband embryo. The germband is severely deformed, with no head lobes and no pre-gnathal segments. The exact identity of the remaining segments is difficult to determine. **(C)** Expression of *hh* in *sgg-*RNAi blastoderm embryo with mild knock-down effect. The expression ring is jagged and not uniform. **(D)** Expression of *hh* in *sgg-*RNAi blastoderm embryo with severe knock-down effect showing patchy expression suggesting that the embryo did not develop at all. Axis labels: A, anterior; P, posterior; D, dorsal; V, ventral. All scale bars are 200 μm.

**FIGURE 5:**
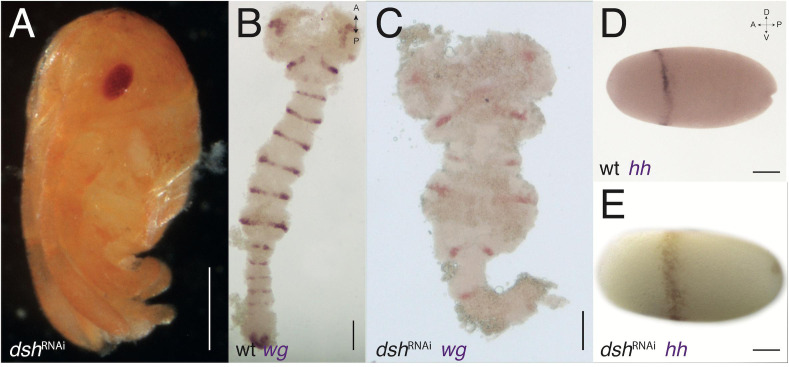
*dsh-*RNAi phenotypes. **(A)***dsh*-RNAi hatchling with no abdomen (compare with Figure 3B). **(B)** Expression of *wg* in wild-type germband stage embryo (slightly older than the one in Figure 2E). **(C)** Expression of *wg* in *dsh-*RNAi germband with normal PGS but deformed gnathal segments and missing thoracic and abdominal segments. **(D)** Expression of *hh* in wild-type blastoderm stage embryo (same embryos from Figure 1A). **(E)** Expression of *hh* in *dsh-*RNAi blastoderm embryo, the anterior ring is jagged and not uniform. Axis labels: A, anterior; P, posterior; D, dorsal; V, ventral. All scale bars are 200 μm.

When increasing the activity of the Wnt pathway *via sgg*^RNAi^ (Figure 4) and decreasing it *via dsh*^RNAi^ (Figure 5), we see opposite and complementary effects. We find that *sgg*^RNAi^ hatchlings have a severely reduced head (Figure 4A). Because the head region is missing in these embryos, and the head is normally the last structure to invaginate (Figures 1L, M) they do not complete invagination properly. It is thus difficult to dissect germband stage *sgg*^RNAi^ embryos, and the mounted germbands are fairly disrupted. Expression of *wg* in in the germband of these embryos is as in WT, but there is no expression in what remains of the anterior segments (Figure 4B). The early expression of *hh* in *sgg*^RNAi^ embryos is diffuse and broader than normal in some embryos (Figure 4C), whereas in others it is completely disrupted (Figure 4D).

In contrast, *dsh*^RNAi^ hatchlings have a reduced abdomen or, in more severe phenotypes, a reduced abdomen and thorax (Figure 5A), and severely disrupted and truncated germbands, with WT appearing head lobes in *dsh*^RNAi^ embryos (Figure 5C compare with Figure 5B). The early expression of *hh* in *dsh*^RNAi^ embryos is broader than normal and is shifted posteriorly compared with WT embryos (Figure 5E compare with Figure 5D). Early expression of *wg* in embryos with the Wnt pathway disrupted also displays a complementary effect. In *sgg*^RNAi^ embryos the anterior expression patch (the “head blob”) is missing, whereas in *dsh*^RNAi^ embryos, the posterior patch is missing (Supplementary Figures 2D, E).

## Discussion

### The Expression of Segment Polarity Genes in the PGS of *Oncopeltus* Is Different From All Other Segments

Previous studies on segment polarity gene expression in *Oncopeltus* (Stahi and Chipman, 2016; Auman et al., 2017) identified two distinct dynamic modes for these genes: segmental stripes of expression of segment-polarity genes appear simultaneously in the gnathal and thoracic segments, and sequentially from a segment addition zone in the abdominal segments. Despite the difference in the early stages of the segmentation cascade, in both cases, *hh* and *inv* are expressed in segmental stripes at the posterior of each segment and *wg* is expressed anterior to them. This expression is the same as that seen in all arthropods studied to date, and presumably reflects the ancient and highly conserved segment-polarity GRN (Von Dassow et al., 2000; Wagner, 2014; Chipman, 2020).

Conversely, as we show here, the expression of the segment polarity genes is different in the PGS. The expression domains of *hh* and *wg* in the ocular segment are different in shape and extent, and do not overlap. The extent and pattern of expression of both these genes in the ocular segment are also different from those of all other segments. Expression of these two genes in the antennal segment is similar, although we were unable to determine whether they overlap. Both genes are expressed in a clear stripe, as in other segments. Expression in the intercalary segment is not in segmental stripes but in two small dots, and these appear later in development, during the germband stage, after all head and thoracic segments are determined and have begun to differentiate. The dynamics of *hh* expression in the PGS are unique, relative to other segment polarity genes in both the PGS and in the rest of the embryo, and involve a stage of a single broad expression stripe splitting to give two stripes. The best studied segment polarity gene in *Oncopeltus*, the transcription factor encoding gene *invected* [an *en* paralog, which presumably shares a similar expression pattern (Peel et al., 2006)] is not expressed in the PGS during the blastoderm stage (Stahi and Chipman, 2016). Its earliest expression is after the formation of the head lobes in the germband.

### The Structure of the Segment-Polarity Network in the PGS in *Oncopeltus* Is Different to All Other Segments

Focusing on the functions of the three aforementioned segment polarity genes and on the interactions among them also identifies key differences between what happens in the PGS and what happens in trunk segments. The expression and function of *inv* in *Oncopeltus* has been previously described (although it was originally misidentified as *en*). Knocking down *inv* leads to trunk segments having distorted segmental borders, while the PGS are unaffected (Angelini and Kaufman, 2005) indicating a difference in the role of this gene between the two groups of segments.

In *hh*^RNAi^ embryos, we find reduced eyes, antennae and labrum, all of which are structures related to the PGS. However, the “classic” segment polarity knock-down phenotype – disruptions to segment boundaries – is not evident. This is most likely because *hh* is induced by *en* in the trunk segments and is part of a regulatory feedback loop. Therefore, a single knockdown may not result in a noticeable effect, as En/Inv can rescue Hedgehog function through compensation. Because we do see a phenotype in the PGS, we suggest that the segment polarity GRN that maintains *hh* expression in the trunk segments is not active in the same way in the PGS.

This idea is strengthened by *ptc*^RNAi^ embryos. The knock down of *ptc* causes over-activation of the pathway and disrupts normal segmental boundaries in the trunk segments only. This is evident in *wg* stained germbands and in the knock-down hatchlings.

In both *dsh*^RNAi^ and *sgg*^RNAi^ embryos we see irregularities in the expression of *hh* during PGS formation. However, only *sgg*^RNAi^ shows a reduction in the head. This is due to the Wnt pathway’s early function in anterior-posterior axis determination of the embryo (Petersen and Reddien, 2009; Stahi and Chipman, 2016). Activation of Wnt in the posterior, early in bilaterian embryogenesis is crucial for proper pole definition. This process is much earlier than PGS formation, therefore the reduction of the head in *sgg*^RNAi^ is probably a result of a posteriorization of the entire embryo following Wnt over-activation. Conversely, *dsh*^RNAi^ embryos show reduction in the posterior pole of the embryo. Mild cases lack the abdomen, while more severe cases lack all segments up until the gnathal segments. In these embryos the head is not affected by the disruption of the axis. However, the gnathal segments show segmental abnormalities and disrupted *wg* expression, while the PGS are normal. This again suggests that the classical segment polarity GRN does not function in the same manner in the PGS.

### The Unique Characteristics of the PGS Are Probably General to All Arthropods

Detailed functional studies as we present here have not been done in many other species. The only example is early work on *Drosophila*, which mostly focused on late stages of PGS patterning and not on their early formation (Gallitano-Mendel and Finkelstein, 1997), but showed that the interactions among the segment-polarity genes in the anterior segments are different from those in other segments. Nonetheless, the non-canonical expression of segment-polarity genes in the anteriormost segments has been shown in many arthropod species, although it has not always been pointed out explicitly. Expression of *en* in the PGS appears later than expected based on their position in myriapods (Kettle et al., 2003; Chipman et al., 2004; Janssen et al., 2004), in spiders (Damen, 2002) and in crustaceans (Scholtz et al., 1994; Hannibal et al., 2012). Stripe splitting of the early *hh* stripe in the PGS (usually the deutocerebral stripe) has been shown in spiders (Kanayama et al., 2011), in myriapods (Janssen, 2012; Hunnekuhl and Akam, 2017), and in insects (Miyawaki et al., 2004; Farzana and Brown, 2008). Expression of *wg* in early patterning of the anterior segments has, been studied in a few insect species, and has been shown to have an early “head blob” expression in the ocular segment, similar to what we have shown (Liu et al., 2006). Comparing the expression pattern of segment polarity genes in the PGS of *Oncopeltus* during the germband stage to those reported in the literature for other insects (see references above and many others) reveals significant similarities – small expression patches for the ocular segment, thick angled antennal stripes, and very small expression patterns for the intercalary segment, starting as two small dots. Despite the patchy nature of the data, the phylogenetic spread of the evidence suggests that there are numerous differences in determination and patterning between the PGS and segments posterior to them in all arthropods. The distinction between the PGS and the trunk is ancient and conserved, and there are several similarities in the patterning of the PGS among different arthropods; most notably, the absence of regulation by pair-rule genes in all three and by Hox genes in the protocerebral and deutocerebral segments, as well as the differences in the segment polarity genes detailed above. In contrast, the interactions among the segment polarity genes in the trunk segments seem to be conserved across arthropods, even under widely differing modes of segmentation (Chipman, 2020).

### The PGS Are Not Serially Homologous to the Trunk Segments

Arthropod segments are said to be serially homologous structures. The exact definition of serial homology has been debated since the early days of comparative morphology (Moment, 1945; Wagner, 2014). While the standard definition of homologous structures is that they are descendent from the same structure in a common ancestor (Minelli and Fusco, 2013), this strict definition cannot be used on structures in the same organism. Thus, we must turn to other definitions of homology. The emerging paradigm within evolutionary developmental biology sees homologous structures as being patterned by conserved GRNs (Wagner, 2007). This definition works for serial homology as well, since several structures in a single organism can be patterned by the same GRN. Wagner (2014) defines serial homology thus: “two body parts of the same organism are serially homologous if they result from the repeated activation of the same *character identity network*” (p. 418, italics added). Similarly, Tomoyasu et al. (2017) identified serially homologous structures as being orchestrated by the same *developmental system*. The conserved GRN (Wagner’s character identity network) underlying serially homologous segments is the segment-polarity gene network. This is the most conserved aspect of the segmentation cascade and is repeatedly activated in almost identical fashion in the formation of all trunk segments in all arthropods studied to date (Peel et al., 2005; Chipman, 2020). We have shown that the three anterior segments of the arthropod head, collectively known as the PGS, do not share this conserved network of interactions, despite the involvement of the same genes in the process. The various components of the segment-polarity network are expressed at different relative times and in different relative positions, and have different functional interactions. We therefore assert that under the definition of serial homology given above, the PGS are not serially homologous to the other segments in the arthropod body. Since many of the unusual aspects of the PGS are shared among different arthropod clades, we believe they are homologous within arthropods, an idea supported by neuroanatomy, by the expression of Hox genes and by the fossil record (Ortega-Hernández et al., 2017).

## Conclusion

We have shown that the GRN patterning the PGS in the insect *Oncopeltus* is fundamentally different from that patterning all other segments. Patchy, but phylogenetically broad data from other arthropods indicates that this is a general phenomenon. We conclude that the three PGS have an evolutionary history that is independent from trunk segments and suggest they may represent the ancestral arthropod head, with the gnathal segments added later in evolution – an idea supported by the fossil record (Ortega-Hernández et al., 2017; Chipman and Edgecombe, 2019). This revives an idea briefly discussed in the late 20th century that the head should be considered to be composed of two tagmata (Minelli, 2001). With this new insight, it should be possible to reinterpret the changes in the morphology of the head throughout arthropod evolution, as represented in the fossil record. This insight also opens the door for more detailed analyses of the development of the head in extant arthropods with the aim of reconstructing the precise changes in developmental regulation that lead to the evolution of the complex head we see today.

## Data Availability Statement

The raw data supporting the conclusions of this article will be made available by the authors, without undue reservation.

## Author Contributions

OL carried out all experimental work, prepared the figures, and wrote the first version of the manuscript. ADC supervised the work and wrote the final version of the manuscript. Both authors contributed to the article and approved the submitted version.

## Conflict of Interest

The authors declare that the research was conducted in the absence of any commercial or financial relationships that could be construed as a potential conflict of interest. The reviewer BCV declared a past collaboration with one of the authors ADC to the handling editor.

## Publisher’s Note

All claims expressed in this article are solely those of the authors and do not necessarily represent those of their affiliated organizations, or those of the publisher, the editors and the reviewers. Any product that may be evaluated in this article, or claim that may be made by its manufacturer, is not guaranteed or endorsed by the publisher.

## References

[B1] Akam M. Dawson I. Tear G. (1988). Homeotic genes and the control of segment diversity. *Development* 104(Suppl.) 123–133. 10.1242/dev.104.supplement.123

[B2] Angelini D. R. Kaufman T. C. (2005). Functional analyses in the milkweed bug *Oncopeltus fasciatus* (Hemiptera) support a role for Wnt signaling in body segmentation but not appendage development. *Dev. Biol.* 283 409–423. 10.1016/j.ydbio.2005.04.034 15939417

[B3] Auman T. Chipman A. D. (2017). The evolution of gene regulatory networks that define arthropod body plans. *Int. Comp. Biol.* 57 523–532. 10.1093/icb/icx035 28957519

[B4] Auman T. Vreede B. M. I. Weiss A. Hester S. D. Williams T. A. Nagy L. M. (2017). Dynamics of growth zone patterning in the milkweed bug *Oncopeltus fasciatus*. *Development* 144 1896–1905. 28432218 10.1242/dev.142091 PMC5450833

[B5] Birkan M. Schaeper N. D. Chipman A. D. (2011). Early patterning and blastodermal fate map of the head in the milkweed bug *Oncopeltus fasciatus*. *Evol. Dev.* 13 436–447. 10.1111/j.1525-142x.2011.00497.x 23016905

[B6] Brown S. J. Patel N. H. Denell R. E. (1994). Embryonic expression of the single *Tribolium Engrailed* Homolog. *Dev. Genet.* 15 7–18. 10.1002/dvg.1020150103 8187351

[B7] Chipman A. D. (2017). *Oncopeltus fasciatus* as an evo-devo research organism. *Genesis* 17:e23020. 10.1002/dvg.23020 28432831

[B8] Chipman A. D. (2020). The evolution of the gene regulatory networks patterning the *Drosophila* blastoderm. *Curr. Top. Dev. Biol* 139 297–324. 10.1016/bs.ctdb.2020.02.004 32450964

[B9] Chipman A. D. Edgecombe G. D. (2019). Developing an integrated understanding of the evolution of arthropod segmentation using fossils and evo-devo. *Proc. Roy. Soc. B* 286:20191881. 10.1098/rspb.2019.1881 31575373 PMC6790758

[B10] Chipman A. D. Arthur W. Akam M. (2004). Early development and segment formation in the centipede *Strigamia maritima* (Geophilomorpha). *Evol. Dev.* 6 78–89. 10.1111/j.1525-142x.2004.04016.x 15009120

[B11] Choe C. P. Miller S. C. Brown S. J. (2006). A pair-rule gene circuit defines segments sequentially in the short-germ insect *Tribolium castaneum*. *Proc. Natl. Acad. Sci. U.S.A.* 103 6560–6564. 10.1073/pnas.0510440103 16611732 PMC1564201

[B12] Clark E. (2017). Dynamic patterning by the *Drosophila* pair-rule network reconciles long-germ and short-germ segmentation. *PLoS Biol.* 15:e2002439. 10.1371/journal.pbio.2002439 28953896 PMC5633203

[B13] Clark E. Peel A. D. Akam M. (2019). Arthropod segmentation. *Development* 19:146. 10.1242/dev.170480 31554626

[B14] Crozatier M. Valle D. Dubois L. Ibnsouda S. Vincent A. (1996). *collier*, a novel regulator of *Drosophila* head development, is expressed in a single mitotic domain. *Curr. Biol.* 6 707–718. 10.1016/s0960-9822(09)00452-7 8793297

[B15] Crozatier M. Valle D. Dubois L. Ibnsouda S. Vincent A. (1999). Head versus trunk patterning in the *Drosophila* embryo; collier requirement for formation of the intercalary segment. *Development* 126 4385–4394. 10.1242/dev.126.19.4385 10477305

[B16] Damen W. G. M. (2002). Parasegmental organization of the spider embryo implies that the parasegment is an evolutionary conserved entity in arthropod embryogenesis. *Development* 129 1239–1250. 10.1242/dev.129.5.1239 11874919

[B17] Damen W. G. Janssen R. Prpic N. M. (2005). Pair rule gene orthologs in spider segmentation. *Evol. Dev.* 7 618–628. 10.1111/j.1525-142x.2005.05065.x 16336415

[B18] Farzana L. Brown S. J. (2008). Hedgehog signaling pathway function conserved in *Tribolium* segmentation. *Dev. Genes Evol.* 218 181–192. 10.1007/s00427-008-0207-2 18392879 PMC2292471

[B19] Gallitano-Mendel A. Finkelstein R. (1997). Novel segment polarity gene interactions during embryonic head development in *Drosophila*. *Dev. Biol.* 192 599–613. 10.1006/dbio.1997.8753 9441692

[B20] Green J. Akam M. (2013). Evolution of the pair rule gene network: insights from a centipede. *Dev. Biol.* 382 235–245. 10.1016/j.ydbio.2013.06.017 23810931 PMC3807789

[B21] Hannibal R. L. Price A. L. Patel N. H. (2012). The functional relationship between ectodermal and mesodermal segmentation the crustacean, *Parhyale hawaiensis*. *Dev. Biol.* 361 427–438. 10.1016/j.ydbio.2011.09.033 22037675

[B22] Hunnekuhl V. S. Akam M. (2017). Formation and subdivision of the head field in the centipede *Strigamia maritima*, as revealed by the expression of head gap gene orthologues and *hedgehog* dynamics. *EvoDevo* 8:18. 10.1186/s13227-017-0082-x PMC5654096 29075435

[B23] Janssen R. (2012). Segment polarity gene expression in a myriapod reveals conserved and diverged aspects of early head patterning in arthropods. *Dev. Genes Evol.* 222 299–309. 10.1007/s00427-012-0413-9 22903234

[B24] Janssen R. Le Gouar M. Pechmann M. Poulin F. Bolognesi R. Schwager E. E. (2010). Conservation, loss, and redeployment of Wnt ligands in protostomes: implications for understanding the evolution of segment formation. *BMC Evol. Biol.* 10:374. 10.1186/1471-2148-10-374 21122121 PMC3003278

[B25] Janssen R. Prpic N.-M. Damen W. G. M. (2004). Gene expression suggests decoupled dorsal and ventral segmentation in the millipede *Glomeris marginata* (Myriapoda: Diplopoda). *Dev. Biol.* 268 89–104. 10.1016/j.ydbio.2003.12.021 15031107

[B26] Kanayama M. Akiyama-Oda Y. Nishimura O. Tarui H. Agata K. Oda H. (2011). Travelling and splitting of a wave of *hedgehog* expression involved in spider-head segmentation. *Nat. Commun.* 2:500. 10.1038/ncomms1510 PMC3207210 21988916

[B27] Kearse M. Moir R. Wilson A. Stones-Havas S. Cheung M. Sturrock S. (2012). Geneious Basic: an integrated and extendable desktop software platform for the organization and analysis of sequence data. *Bioinformatics* 28 1647–1649. 10.1093/bioinformatics/bts199 22543367 PMC3371832

[B28] Kettle C. Johnstone J. Jowett T. Arthur H. Arthur W. (2003). The pattern of segment formation, as revealed by *engrailed* expression, in a centipede with a variable number of segments. *Evol. Dev.* 5 198–207. 10.1046/j.1525-142x.2003.03027.x 12622737

[B29] Liu Z. Yang X. Dong Y. Friedrich M. (2006). Tracking down the “head blob”: comparative analysis of *wingless* expression in the developing insect procephalon reveals progressive reduction of embryonic visual system patterning in higher insects. *Arthropod. Struct. Dev.* 35 341–356. 10.1016/j.asd.2006.07.003 18089080

[B30] Minelli A. (2001). A three-phase model of arthropod segmentation. *Dev. Genes.Evol.* 211 509–521. 10.1007/s004270100180 11702202

[B31] Minelli A. Fusco G. (2013). “Homology,” in *The Philosophy of Biology: A Companion for Educators*, ed. Kampourakis K. (Dordrecht: Springer), 289–322.

[B32] Miyawaki K. Mito T. Sarashina I. Zhang H. J. Shinmyo Y. Ohuchi H. (2004). Involvement of *Wingless/Armadillo* signaling in the posterior sequential segmentation in the cricket, *Gryllus bimaculatus* (Orthoptera), as revealed by RNAi analysis. *Mech. Dev.* 121 119–130. 10.1016/j.mod.2004.01.002 15037314

[B33] Moment G. B. (1945). The relationship between serial and special homology and organic similarities. *Am. Nat.* 79 445–455. 10.1086/281279

[B34] Murat S. Hopfen C. Mcgregor A. P. (2010). The function and evolution of Wnt genes in arthropods. *Arthropod. Struct. Dev.* 39 446–452. 10.1016/j.asd.2010.05.007 20685345

[B35] Nagy L. M. Williams T. A. (2020). “Cell division, movement and synchronization in arthropod segmentation,” in *Cellular Processes in Segmentation*, ed. Chipman A. D. (Boca Raton, FL: CRC Press), 40–70.

[B36] Nüsslein-Volhard C. Wieschaus E. (1980). Mutations affecting segment number and polarity in *Drosophila*. *Nature* 287 795–801. 10.1038/287795a0 6776413

[B37] Ortega-Hernández J. Janssen R. Budd G. E. (2017). Origin and evolution of the panarthropod head–A palaeobiological and developmental perspective. *Arthropod. Struct. Dev.* 46 354–379. 10.1016/j.asd.2016.10.011 27989966

[B38] Patel N. H. Kornberg T. B. Goodman C. S. (1989). Expression of *engrailed* during segmentation in grasshopper and crayfish. *Development* 107 201–212. 10.1242/dev.107.2.201 2632219

[B39] Pechmann M. Mcgregor A. P. Schwager E. E. Feitosa N. M. Damen W. G. M. (2009). Dynamic gene expression is required for anterior regionalization in a spider. *Proc. Natl. Acad Sci. U.S.A.* 106 1468–1472. 10.1073/pnas.0811150106 19147844 PMC2635816

[B40] Peel A. D. Chipman A. D. Akam M. (2005). Arthropod segmentation: beyond the *Drosophila* paradigm. *Nat. Rev. Genet.* 6 905–916. 10.1038/nrg1724 16341071

[B41] Peel A. D. Telford M. J. Akam M. (2006). The evolution of hexapod *engrailed*-family genes: evidence for conservation and concerted evolution. *Proc. Biol. Sci.* 273 1733–1742. 10.1098/rspb.2006.3497 16790405 PMC1634793

[B42] Petersen C. P. Reddien P. W. (2009). Wnt signaling and the polarity of the primary body axis. *Cell* 139 1056–1068. 10.1016/j.cell.2009.11.035 20005801

[B43] Posnien N. Schinko J. B. Kittelmann S. Bucher G. (2010). Genetics, development and composition of the insect head–A beetle’s view. *Arthropod. Struct. Dev.* 39 399–410. 10.1016/j.asd.2010.08.002 20800703

[B44] Reding K. Chen M. Lu Y. Cheatle Jarvela A. M. Pick L. (2019). Shifting roles of *Drosophila* pair-rule gene orthologs: segmental expression and function in the milkweed bug *Oncopeltus fasciatus*. *Development* 19:146. 10.1242/dev.181453 PMC6765130 31444220

[B45] Rogers B. T. Kaufman T. C. (1997). Structure of the insect head in ontogeny and phylogeny: a view from *Drosophila*. *Int. Rev. Cytol.* 174 1–84. 10.1016/s0074-7696(08)62115-4 9161005

[B46] Scholtz G. (2020). “Segmentation: a zoological concept of seriality,” in *Cellular Prcesses in Segmentation*, ed. Chipman A. D. (Boca Raton, FL: CRC Press), 3–25. 10.1201/9780429423604-2

[B47] Scholtz G. Patel N. H. Dohle W. (1994). Serially homologous *engrailed* stripes are generated via different cell lineages in the germ band of amphipod crustaceans (Malacostraca, Peracarida). *Int. J. Dev. Biol.* 38 471–478. 7848831

[B48] Stahi R. Chipman A. D. (2016). Blastoderm segmentation in *Oncopeltus fasciatus* and the evolution of arthropod segmentation mechanisms. *Proc. R. Soc. Lond. B* 283:20161745. 10.1098/rspb.2016.1745 27708151 PMC5069518

[B49] Tomoyasu Y. Ohde T. Clark-Hachtel C. (2017). What serial homologs can tell us about the origin of insect wings. *F1000Research* 6:268. 10.12688/f1000research.10285.1 28357056 PMC5357031

[B50] Von Dassow G. Meir E. Munro E. M. Odell G. M. (2000). The segment polarity network is a robust developmental module. *Nature* 406 188–192. 10.1038/35018085 10910359

[B51] Wagner G. P. (2007). The developmental genetics of homology. *Nat. Rev. Genet.* 8 473–479. 10.1038/nrg2099 17486120

[B52] Wagner G. P. (2014). *Homology, Genes and Evolutionary Innovation.* Oxford: Princeton University Press.

[B53] Wimmer E. A. Cohen S. M. Jackle H. Desplan C. (1997). *buttonhead* does not contribute to a combinatorial code proposed for *Drosophila* head development. *Development* 124 1509–1517. 10.1242/dev.124.8.1509 9108367

