# Supplementary figures and images for: Development of the Pre-gnathal Segments in the Milkweed Bug Oncopeltus fasciatus Suggests They Are Not Serial Homologs of Trunk Segments
Source: Front Cell Dev Biol. 2021 Aug 6;9:695135. doi: 10.3389/fcell.2021.695135 (PMC8378449; doi:10.3389/fcell.2021.695135)

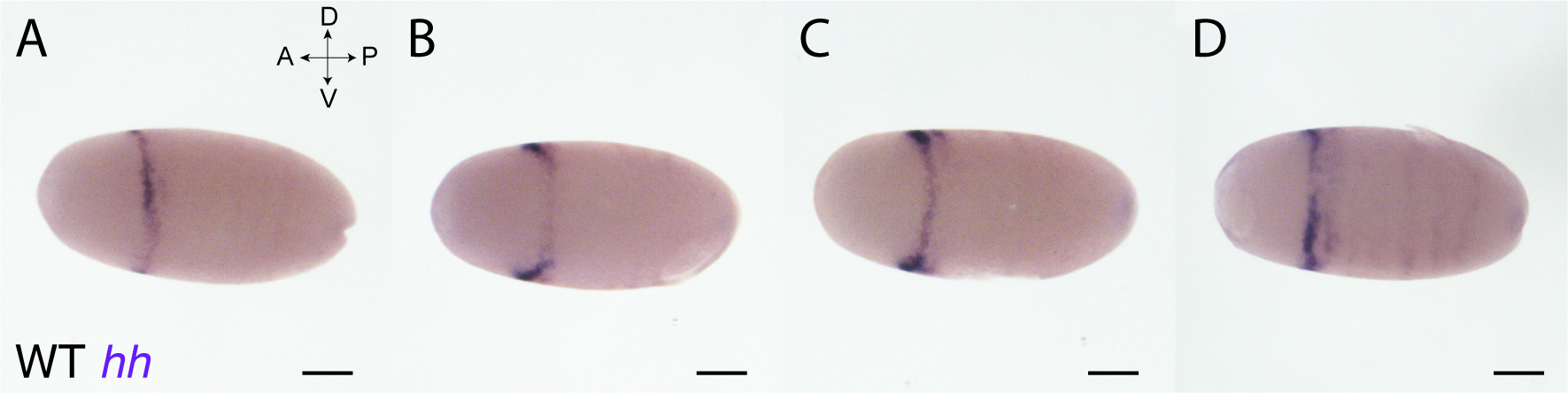

Supplement: Supplementary file 1 [file Image_1.TIF]

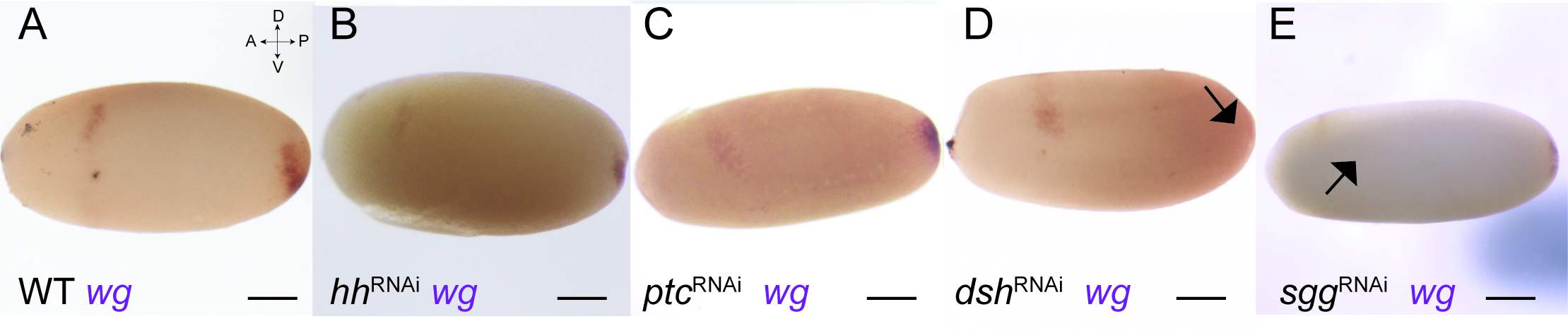

Supplement: Supplementary file 2 [file Image_2.TIF]
