# Supplementary material for: Development of the Pre-gnathal Segments in the Milkweed Bug Oncopeltus fasciatus Suggests They Are Not Serial Homologs of Trunk Segments
Source: Front Cell Dev Biol. 2021 Aug 6;9:695135. doi: 10.3389/fcell.2021.695135 (PMC8378449; doi:10.3389/fcell.2021.695135)
Supplement: Supplementary file 3 [file Data_Sheet_1.DOCX]

Sequences of primers used to prepare DIG-labeled probes:

|  | Forward Primer | Reverse Primer | GenBank accession number: |
| --- | --- | --- | --- |
| *hedgehog* | CTAGCGGTGGTCTGTTGGG | GTTTCAGCAACGCTCACTTCT | MH090996 |
| *wingless* | CTTCTGCGAGAGGAATCCAAGG | AGGCCCTGAAGTCGATCATTAAT | AY899335 |
| *patched* | AGACAACCCAATTGACCGATCA | AGCCACCAAAGCTAAACCCA | MW008845 |
| *disheveled* | TTCTTGGCTTGTTTCTGCAGATG | TCCCACAATGCTGATACCAAGAA | MW008846 |
| *shaggy* | TCCACACATCGATTTTGGTTGTG | TTAAGAACCGGGAGCTTCAGATC | MW008847 |

Sequences of primers used to prepare doubles stranded RNA for parental RNA interference:

|  | Forward Primer | Reverse Primer |
| --- | --- | --- |
| *hedgehog* 1^st^ fragment | TAATACGACTCACTATAGGGTACTACGAGACCAGAGCGC | TAATACGACTCACTATAGGGTCCTCGCAGCAAAGACAGTT |
| *hedgehog* 2^nd^ fragment | TAATACGACTCACTATAGTTCAAGCAGCACGTACCCAA | TAATACGACTCACTATAGGCCAACATCCCGTACTTGGA |
| *patched* 1^st^ fragment | TAATACGACTCACTATAGGGAAGCAGATCAACAAGGGAAAAGC | TAATACGACTCACTATAGGGCCCAAAAACAATCCAGAGGTGTC |
| *patched* 2^nd^ fragment | TAATACGACTCACTATAGGGCTTGGGTTTTGTGCTGTCTTAGG | TAATACGACTCACTATAGGGAGGAAGGGCTCTCGTAATTGTTT |
| *disheveled* 1^st^ fragment | TAATACGACTCACTATAGGGTTCTTGGCTTGTTTCTGCAGATG | TAATACGACTCACTATAGGGTCCCACAATGCTGATACCAAGAA |
| *disheveled* 2^nd^ fragment | TAATACGACTCACTATAGGGTTCTTGGTATCAGCATTGTGGGA | TAATACGACTCACTATAGGGTGGAGGAACGTAACCATATAGCG |
| *shaggy* 1^st^ fragment | TAATACGACTCACTATAGGGCAGTTCGTCAAAGAAGGAATGGG | TAATACGACTCACTATAGGGCACAACCAAAATCGATGTGTGGA |
| *shaggy* 2^nd^ fragment | TAATACGACTCACTATAGGGACGTTTGGTTCGCCTTTGATAAG | TAATACGACTCACTATAGGGTTAAGAACCGGGAGCTTCAGATC |
