# Supplementary material for: Development of the Pre-gnathal Segments in the Milkweed Bug Oncopeltus fasciatus Suggests They Are Not Serial Homologs of Trunk Segments
Source: Front Cell Dev Biol. 2021 Aug 6;9:695135. doi: 10.3389/fcell.2021.695135 (PMC8378449; doi:10.3389/fcell.2021.695135)
Supplement: Supplementary file 4 [file Data_Sheet_2.DOCX]

**Supplementary legend**

Supplementary Figure 1 – The development of wildtype expression of *hedgehog* in blastoderm embryos. A-D) from left to right: embryos from ~30 hours after egg laying (hAEL) to ~36 hAEL. an anterior ring of *hh* expression appears ~30 hAEL, which splits gradually, resulting in two stripes correlating to the ocular and antennal segments. the splitting continues during invagination, which starts at ~36 hAEL.

Supplementary Figure 2 – Expression of *wingless* in embryos ~32 hours after egg laying (hAEL) following pRNAi against different genes. (A) wildtype expression of *wg* is in an anterior patch which will be the ocular staining (“head blob”), and a posterior patch marking the future site of invagination. (B) Expression of *wg* in *hh*-RNAi embryos shows no significant difference from wildtype. (C) Expression of *wg* in *ptc*-RNAi embryos shows no significant difference from wildtype. (D) Expression of *wg* in *dsh*-RNAi embryos is missing in the posterior future site of invagination (black arrow), the anterior patch is the same as in wildtype embryos. (E) Expression of *wingless* in *sgg*-RNAi embryos is missing the anterior ocular staining (black arrow), the posterior patch is the same as in wildtype embryos.
